# Prevalence, incidence, and years lived with disability due to Autism Spectrum Disorder in Brazil, 1990-2023: Results from the Global Burden of Disease Study 2023

**DOI:** 10.1590/1980-549720260025

**Published:** 2026-08-07

**Authors:** Nathalia Avila Dimer, Ana Paula Souto Melo, Damian Santomauro, Deborah Carvalho Malta, Bruce Bartholow Duncan, Bárbara Niegia Garcia de Goulart

**Affiliations:** I Universidade Federal do Rio Grande do Sul, Postgraduate Program in Epidemiology - Porto Alegre (RS), Brazil.; II Universidade Federal de Minas Gerais, Postgraduate Program in Public Health - Belo Horizonte (MG), Brazil.; III Universidade Federal de São João del-Rei, School of Medicine, Department of Psychiatry - São João del-Rei (MG), Brazil.; IV University of Washington, Institute for Health Metrics and Evaluation - Seattle (WA), USA.; V Universidade Federal de Minas Gerais, Department of Maternal-Child Nursing and Public Health - Belo Horizonte (MG), Brazil.

**Keywords:** Autism Spectrum Disorder, Global burden of disease, Epidemiology, Prevalence, Incidence, Disability-adjusted life years, Transtorno do Espectro Autista, Carga global da doença, Epidemiologia, Prevalência, Incidência, Anos de vida ajustados por deficiência

## Abstract

**Objective:**

To describe the prevalence, incidence, and years lived with disability due to autism spectrum disorder in Brazil, as well as the variation of these metrics between 1990 and 2023.

**Methods:**

A time-series analysis using data for Brazil from the 2023 Global Burden of Disease study. Estimates of incidence, prevalence, and years lived with disability attributable to autism spectrum disorder were generated for Brazil and its regions. The study reports absolute frequencies, crude rates, and age-standardized rates per 100,000 inhabitants, all with 95% uncertainty intervals (95% UI), stratified by sex and age group.

**Results:**

In 2023, more than 1.1 million (95% UI: 0.58; 1.97) individuals on the autism spectrum were living in Brazil, representing 0.53% of the population (95% UI: 0.27; 0.93). The number of cases increased since 1990, while age-standardized prevalence remained stable over time. In 2023, more than 23,000 new autism spectrum disorder cases were estimated, with a rate of 11.19 per 100,000 individuals (95% UI: 2.47-31.39). Autism spectrum disorder was ranked within the top ten causes of years lived with disability for people younger than 20 years, being a non-fatal health burden.

**Conclusion:**

In 2023, 0.53% of Brazilians were autistic, corresponding to more than 207,000 years lived with disability. Prevalence was higher in males and children under five years of age. The permanent and complex nature of autism spectrum disorder imposes a significant burden, requiring healthcare and education services to be adequately prepared. Further research is needed to deepen the understanding of autism spectrum disorder and validate screening tools in the Brazilian context.

## RESUMO

**Objetivo:**

Descrever a prevalência, a incidência e os anos vividos com incapacidade (YLDs) por conta do transtorno do espectro autista (TEA) no Brasil, bem como sua variação entre 1990 e 2023.

**Métodos:**

Estudo de série temporal no Brasil, com dados do Estudo da Carga Global de Doenças (GBD) 2023. Foram estimadas incidência, prevalência e YLDs atribuídos ao TEA no Brasil e em suas regiões. O estudo apresenta frequências absolutas, taxas brutas e ajustadas por idade por 100 mil habitantes, com intervalos de incerteza de 95% (95% II), estratificadas por sexo e faixa etária.

**Resultados:**

Em 2023, mais de 1,1 milhão (II 95% 0,58; 1,97) de pessoas no espectro do autismo viviam no Brasil, representando 0,53% da população (II 95% 0,27; 0,93). O número de casos aumentou desde 1990, enquanto a prevalência ajustada por idade permaneceu estável ao longo do tempo. Em 2023, estimaram-se mais de 23 mil novos casos de TEA, com taxa de 11,19 por 100 mil (II 95% 2,47; 31,39). O TEA foi classificado entre as dez principais causas de YLDs por problemas de saúde não fatais em pessoas com menos de 20 anos.

**Conclusão:**

Em 2023, 0,53% dos brasileiros eram autistas, correspondendo a mais de 207 mil anos vividos com incapacidade. A prevalência foi maior em homens e em crianças menores de cinco anos. A natureza permanente e complexa do TEA impõe uma carga significativa, exigindo preparo adequado dos serviços de saúde e educação. São necessárias mais pesquisas para aprofundar a compreensão do TEA e validar ferramentas de triagem no contexto brasileiro.

## INTRODUCTION

Autism spectrum disorder (ASD) is a neurodevelopmental condition emerging in early childhood^1^. It is characterized by challenges in social interaction, communication, and sensorimotor behaviors, leading to restricted, repetitive behaviors and interests. For diagnosis, symptoms must cause impairment in daily functioning to establish the diagnosis^1^.

The age at which functional impairment becomes apparent varies. It depends on individual traits and environmental factors^1^. The manifestations of ASD also differ between people and throughout a lifetime. Some individuals with ASD live independently, while others have severe disabilities and need ongoing support^2^ ^,^ ^3^. Children and adolescents with ASD need assistance in behavioral, educational, health, recreational, and family support areas. These needs impact the mental health and financial well-being of individuals, families, and society^2^ ^,^ ^3^.

People with ASD worldwide often experience stigmatization, discrimination, and human rights violations. Access to services and support for these individuals is often lacking. Individuals with ASD have higher rates of depression, anxiety, sleep difficulties, and epilepsy than people without ASD. However, early and continuing interventions can improve development and well-being^4^. Timely evidence-based psychosocial interventions help autistic children communicate and interact socially^5^.

Globally, about 1 in 127 children is autistic^5^, according to estimates from the World Health Organization (WHO), with substantial sociodemographic variation. A 2018 study in southern Brazil found a 0.3% prevalence of ASD in 11-year-olds^6^. In Brazil, the burden of ASD on the Unified Health System is substantial. A study of consultations in the Child and Adolescent Psychosocial Care Center (2013-2019) found 31% of users had ASD^7^.

In addition, substantial additional costs associated with caring for children with ASD are estimated. A literature review published in 2014 estimated that the lifetime cost of supporting a person with ASD and intellectual disability was US$2.4 million in the United States and £1.5 million (equivalent to US$2.2 million) in the United Kingdom. For individuals with ASD without intellectual disabilities, the cost was $1.4 million in the United States and £0.92 million ($1.4 million) in the United Kingdom. Among children, the main cost drivers were special education services and lost parental productivity. In adulthood, the highest expenses were associated with residential care or assisted living, as well as productivity loss. Furthermore, medical costs were significantly higher for adults than for children^8^.

Individuals with neurodevelopmental challenges, including ASD, have benefited from policies promoting inclusion in healthcare, social, and educational systems. However, it is difficult to adapt these policies without accurate estimates across all age groups. To prepare healthcare, education, and the economy to serve this population, it is crucial to measure ASD prevalence, incidence, and years living with disability (YLDs) in Brazil, by sex, age, and region.

### Objective

To describe the prevalence, incidence, and years lived with disability due to ASD in Brazil, as wella as the variation of these metrics between 1990 and 2023.

## METHODS

We conducted a time-series study of ASD in Brazil, using data from the 2023 Global Burden of Disease (GBD) Study.

### Definitions

ASD is a lifelong neurodevelopmental condition. ASD is characterized by global impairment in several areas, including social interaction, communication, and restricted or repetitive behaviors, interests, and activities. To establish the diagnosis, symptoms must be present early, cause clinically significant impairment, and not be better explained by intellectual impairment or global developmental delay.

ASD covers five sub-disorders in the fourth edition of the Diagnostic and Statistical Manual of Mental Disorders (DSM-4): autistic disorder (299.00), pervasive developmental disorder, pervasive developmental disorder not otherwise specified (299.80), Rett’s disorder (299.8), Asperger’s disorder (299.8), and childhood disintegrative disorder (299.10)^9^. In DSM-5, these were grouped into a single disorder^1^.

ASD is also an umbrella term for eight sub-disorders in the International Statistical Classification of Diseases and Related Health Problems, Tenth Revision (ICD-10): childhood autism (F84.0), atypical autism (F84.1), Rett syndrome (F84.2), other childhood disintegrative disorders (F84.3), hyperactive disorder with mental retardation and stereotyped movements (F84.4), Asperger syndrome (F84.5), other global developmental disorders (F84.8), and global disorder not otherwise specified (F84.9)^10^.

The method for estimating the prevalence, incidence, and YLDs of ASD has been detailed previously^11^. First, epidemiological data were obtained through systematic reviews and consultations with experts to verify data validity.

We estimated prevalence using DisMod-AT and informed the model with data meeting the inclusion criteria described below. DisMod-AT is a Bayesian disease modeling meta-regression tool. It generates internally consistent estimates of prevalence, incidence, remission, and mortality by sex, location, year, and age group. The GBD Study 2023 also assumed that all ASD incidence occurred at birth. The proportion of births to mothers aged 35 years or older was used as a location-level covariate for incidence, and the healthcare access quality index was used as a location-level covariate for excess mortality.

The disability weights for each ASD sequela were calculated using the disability weights for the following health states: autism; Asperger syndrome and other ASDs; borderline intellectual disability; mild intellectual disability; moderate intellectual disability; severe intellectual disability; and profound intellectual disability. These disability weights and their lay descriptions can be found in Table S1 of the Supplementary Material ^11^. Disability-adjusted life-years (DALYs) are the aggregate of two health metrics: years lived with disability (YLD), which capture the disability (non-fatal impact) of a cause; and years of life lost (YLL), which capture the fatal impact of a cause. Given that there were no deaths attributable to ASD as an underlying cause in GBD Study 2023 and, therefore, DALYs were composed entirely of YLDs, in this article, we will use YLD as the main metric, since its values are the same as for DALY^1^ ^,^ ^10^ ^,^ ^11^.

### Data source

The study used data from two systematic reviews conducted in 2017 and 2021. In Brazil, the GBD Study included only two data sources for estimates of ASD. As inclusion criteria, the studies considered should have been conducted as of 1980, using diagnostic criteria based on the DSM (DSM-III, DSM-III-R, DSM-IV, DSM-IV-TR, DSM-5, and DSM-5-TR), the ICD (ICD-9, ICD-10, and ICD-11), the Chinese Classification of Mental Disorders (CCMD), or diagnoses made by physicians using recognized tools. Additionally, studies had to provide sufficient information about their methods and sample characteristics to enable quality assessment. Only samples representative of the general population were included, excluding case studies and those based on specific populations, such as veterans or refugees. The detailed characteristics of the studies used by the GBD Study are described in Table S2 ^11^.

### Data Analysis

We present estimates of the burden of ASD in Brazil and in each of its regions, considering sex and age group. We describe the incidence, prevalence, and YLDs caused by ASD. The results were outlined using absolute frequency, crude rates, and age-adjusted rates, expressed as numbers per 100,000 inhabitants, with 95% uncertainty intervals (UI).

## Data availability statement:

The dataset that supports the findings of this study is publicly available from the Global Burden of Disease (GBD) study at https://vizhub.healthdata.org/gbd-results/.

## RESULTS

In 2023, it is estimated that there were more than 1.1 million (95% UI: 0.58; 1.97) autistic people in Brazil, corresponding to 0.53% (95% UI: 0.27; 0.93) of the Brazilian population. There has been an increase in the number of cases compared to 1990, when there were 759,860 (95%UI 330,01-1,696,99) individuals with ASD, totaling 0.51% (95%UI 0.22-1.14) of the population. Between 1990 and 2023, the age-standardized prevalence of ASD remained stable, with a variation rate of 0.03 (95%UI -0.39-1.04). The age-standardized prevalence rates per 100,000 inhabitants were 557.33 (95%UI 280.03-1002.72) in 2023 and 477.1 (95%UI 206.65-1063.57) in 1990, with a variation rate of 0.03 (95%UI -0.39-1.05) (Table 1).

**Table 1.** Prevalence, incidence, and years lived with disability due to Autism Spectrum Disorder in 1990 and 2023 in Brazil.

	Counts (N) (95% UI)		Crude Rate (95% UI)			Age Std Rate (95% UI)		
	1990 N	2023 N	1990 (/100,000 population)	2023 (/100,000 population)	Rate of change	1990 (/100,000 population)	2023 (/100,000 population)	Rate of change
Prevalence	759862.03 (330010.94 ; 1696985.13)	1113425.96 (576753.07 ; 1972912.6)	508.84 (220.99 ; 1136.39)	525 (271.95 ; 930.26)	0.03 (-0.39 ; 1.04)	477.1 (206.65 ; 1063.57)	557.33 (280.03 ; 1002.72)	0.17(-0.39 ; 1.58)
Incidence	19351.02 (9717.91 ; 36981.1)	23723.94 (5808.13 ; 66569.85)	12.96 (6.51 ; 24.76)	11.19 (2.74 ; 31.39)	-0.14(-0.78 ; 2.01)	12.08 (6.07 ; 23.09)	16.68 (4.08 ; 46.82)	0.38(-0.65 ; 3.81)
YLDs	142459.01 (58296.4 ; 300696.55)	207940.33 (97550.56 ; 396404.48)	95.4 (39.04 ; 201.36)	98.05 (46 ; 186.91)	0.03(-0.41 ; 1.06)	89.02 (36.04 ; 188.63)	104.93 (48.53 ; 215.6)	0.18(-0.4 ; 1.64)

Std: standardized.

In addition, in 2023, more than 23,000 (95%UI 5.80-66.57) new cases of ASD were identified, or 11.19 (95%UI 2.74-31.39) cases per 100,000 inhabitants. In 1990, an estimated 19,350 (95%UI 9.72-36.98) new cases of ASD, or 12.96 (95%UI 6.51-24.76) cases per 100,000 inhabitants, indicating a slight decrease in the incidence rate of -0.14 (95%UI -0.78-2.01) between 1990 and 2023.

ASD was the sixth leading cause of YLDs among mental disorders in Brazil in 2023, accounting for 207,940 YLDs (95%UI 97,55-396,40) in the country (98.05 YLDs per 100,000 inhabitants, 95%UI 46.00-186.91). In 1990, it was considered the 5th leading cause of YLDs among mental disorders in the country, accounting for approximately 142,460 YLDs (95%UI 58.30-300.70), (95.40 YLDs per 100,000 inhabitants, 95%UI 39.04-201.36). In 2023, ASD accounted for 0.7% (95%UI 0.3-1.31) of total YLDs for all ages due to mental disorders and 4.06% (1.0; 13.33) in children under 5 years of age. In 1990, ASD accounted for 0.89% (0.34; 1.88) of total YLDs for mental disorders across all ages and 2.73% (1.03; 5.43) in children under 5 years of age.

**Figure 1.**
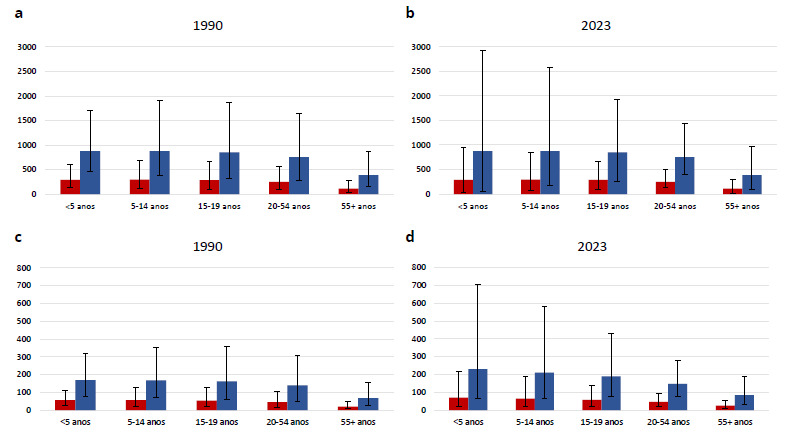
Prevalence rate per 100,000 individuals (“a” and “b”) and rate of years lived with disability per 100,000 individuals (“c” and “d”) for Autism Spectrum Disorder in 1990 and 2023 in Brazil, with their respective 95% uncertainty intervals, stratified by sex (red for women and blue for men) and age group.

At all ages, ASD was more prevalent in men than in women (Figure 1). In 2023, ASD affected 0.82% (95%UI 0.43-1.42) of men of all ages and 0.25% of women (95%UI 0.12-0.47). In 1990, it affected 0.77% (95%UI 0.34-1.68) of men and 0.25% of women (0.22; 1.14). Furthermore, in 2023, there were more new cases in men than in women, with 18,373.25 (95%UI 4,550.22-51,252.82) and 5,350.69 (95%UI 1,257.91-15,317.03), respectively.

In 2023, among men, ASD accounted for 5.89% (95%UI 1.55-17.32) of total YLDs in children under 5 years of age and 1.24% (95%UI 0.854-2.31) of total YLDs in all ages. For women, ASD accounted for 1.98% (95%UI 0.46-6.25) of total YLDs in children under 5 and 0.29% (95%UI 0.12-0.56) of total YLDs across all ages. In 1990, ASD accounted for 0.41% (95%UI 0.14-0.86) of total YLDs among women of all ages and 1.37% (95%UI 0.5-2.88) in children under 5 years of age. For men, ASD accounted for 1.5% (95%UI 0.58-3.1) of total YLDs across all ages and 4% (95%UI 1.5-7.82) in children under 5 years of age.

The region with the highest crude rate of cases per 100,000 inhabitants in 2023 is the Northeast, with 537.36 cases per 100,000 inhabitants (95%UI: 273.34-967.16), while the region with the lowest rate per 100,000 inhabitants is the Midwest (508.57, 95%UI 261.16-901.46). Furthermore, when standardized by age, the South region has the highest prevalence, with 568.98 cases per 100,000 inhabitants (95%UI 297.99-1009.05). The incidence rate for all ages was highest in the Northeast region (15.29; 95%UI 3.73-43.01) and lowest in the South region (8.97; 95%UI 2.28-24.74) (Table 2).

**Table 2.** Crude and age-adjusted prevalence and incidence rates of Autism Spectrum Disorder in 2023 (per 100,000 inhabitants), and variation from 1990 to 2023 in Brazil.

	Prevalence (95% UI)				Incidence (95% UI)			
	2023	1990-2023	2023	1990-2023	2023	1990-2023	2023	1990-2023
	Crude rate (/100,000 population)	Crude Rate of change (/100,000 population) (1990-2023)	Age Std Rate (/100,000 population)	Age Std Rate of change (/100,000 population) (1990-2023)	Crude rate (/100,000 population)	Crude Rate of change (/100,000 population) (1990-2023)	Age Std Rate (/100,000 population)	Age Std Rate of change (1990-2023) (/100,000 population)
Brazil	525 (271.95 ; 930.26)	0.03 (-0.39 - 1.04)	557.33 (280.03 ; 1002.72)	0.17 (-0.39 - 1.58)	11.19 (2.74 ; 31.39)	-0.14 (-0.78 - 2.01)	16.68 (4.08 ; 46.82)	0.38 (-0.65 - 3.81)
Northern Region	526.68 (269.12 ; 955.1)	0.03 (-0.44 - 1.16)	526.45 (263.77 ; 953.48)	0.14 (-0.4 - 1.51)	12.58 (3.07 ; 35.36)	-0.08 (-0.77 - 2.19)	15.32 (3.74 ; 43.07)	0.36 (-0.65 - 3.74)
Northeastern Region	537.36 (273.34 ; 967.16)	0.03 (-0.41 - 1.11)	558.85 (279.3 ; 1008.24)	0.16 (-0.39 - 1.57)	15.29 (3.73 ; 43.01)	-0.02 (-0.75 - 2.39)	17.16 (4.18 ; 48.25)	0.39 (-0.65 - 3.81)
Central-Western Region	508.57 (261.16 ; 901.46)	0.04 (-0.4 - 1.07)	531.62 (264.9 ; 955.75)	0.17 (-0.39 - 1.6)	9.73 (2.37 ; 27.39)	-0.21 (-0.8 - 1.74)	15.69 (3.82 ; 44.14)	0.39 (-0.65 - 3.82)
Southern Region	526.85 (286.57 ; 973.34)	0.03 (-0.37 - 1)	568.98 (297.99 ; 1009.05)	0.17 (-0.38 - 1.59)	8.97 (2.28 ; 24.74)	-0.22 (-0.8 - 1.76)	16.52 (4.19 ; 45.57)	0.36 (-0.65 - 3.82)
Southeastern Region	519.02 (269.03 ; 945.23)	0.03 (-0.38 - 1.02)	565 (281.34 ; 1020.3)	0.19 (-0.38 - 1.63)	9.26 (2.25 ; 26.04)	-0.2 (-0.8 - 1.77)	16.87 (4.11 ; 47.45)	0.39 (-0.65 - 3.85)

Std: standardized.

**Figure 2.**
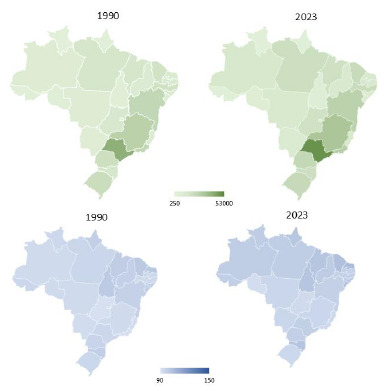
Cases of Autism Spectrum Disorder (green) and crude rate per 100,000 individuals (blue) of years lived with this disability in Brazil in 1990 and 2023.

In 2023, the highest number of YLDs is in the Southeast region (85,101.26, 95%UI 39,638.09-160,225.64), while the highest crude rate is in the Northeast region (100.63, 95%UI 46.48-196.80) (Figure 2). The region with the lowest number and rate of YLDs is the Midwest.

## DISCUSSION

In this study of ASD in Brazil and its regions, based on data from the GBD Study 2023, the estimated prevalence of ASD in the Brazilian population is 0.53% in 2023. An incidence of 23,000 new cases was observed in 2023, with a higher prevalence in males under 5 years of age. Between 1990 and 2023, the crude incidence rate for ASD decreased, the crude prevalence rate remained stable, and the crude YLD rate increased.

While the GBD Study 2023 estimates the prevalence of ASD in Brazil at around 0.53%, the 2022 Brazilian Census identified that 1.2% of the population self-identifies as autistic. This discrepancy suggests that the GBD Study model may underestimate cases, since a case is only confirmed if the diagnosis was made by a medical evaluation using recognized tools. On the other hand, the Census may overestimate prevalence due to response biases and variation in access to clinical diagnosis, since information on ASD was obtained through self-reported diagnoses made by a healthcare professional^12^.

In addition, the Census also identified that ASD affects men more (1.5% of men and 0.9% of women) and is more prevalent in younger age groups (2.1% in the 0-4 age group, 2.6% in the 5-9 age group, 1.9% among those aged 10 to 14, and 1.3% among those aged 15 to 19)^12^.

A systematic review published in 2022, which included 71 cross-sectional studies from 34 countries, estimated the global prevalence of ASD to be approximately 1%, with substantial variation within and between sociodemographic groups⁵. In line with these findings, estimates from the United States also demonstrate considerable variability. Using epidemiological surveillance data from the Autism and Developmental Disabilities Monitoring Network, the US Centers for Disease Control and Prevention applied hierarchical Bayesian modeling with restricted maximum likelihood random-effects methods to estimate ASD prevalence among 8-year-old children. In 2022, the estimated prevalence in this age group ranged from 0.97% to 5.31%^13^.

In this sense, the prevalence of ASD in Brazil seems slightly lower than that found globally. However, given that the studies included in the systematic review are mostly from developed countries, it is expected that the prevalence is higher due to lower stigma and greater availability of and access to healthcare services in more developed countries^14^ ^,^ ^15^ ^,^ ^16^. Globally, knowledge about autism has advanced substantially. Since 2012, the WHO has promoted global and coordinated efforts for the management of ASD. However, these advances have not always resulted in a significant increase in the provision of specialized services^17^ ^,^ ^18^.

The incidence of ASD in Brazil in 2023 was 11.19 new cases per 100,000 inhabitants. This finding contrasts with data from studies, such as the one conducted in the United Kingdom between 2004 and 2010, which involved 8-year-old children. In this study, annual incidence rates of approximately 120/100,000 for boys and 20/100,000 for girls were found^19^. However, a study conducted in China identified a higher incidence rate than that observed in our analysis, possibly due to the age group of the individuals included, in which the diagnosis is more frequent^20^. Importantly, these studies estimate incidence based on the moment of diagnosis, whereas the GBD Study calculates incidence according to the individual’s year of birth, considering that ASD is a lifelong condition.

However, a study conducted with data from 2013 to 2019 in Brazil, using outpatient information, points out that although the incidence remains stable and the prevalence decreases, diagnoses are occurring earlier^7^. It is well established that early diagnosis offers a greater opportunity for intervention and enhances the quality of life for these individuals. However, it also means a greater demand for interventions, and consequently, requires more planning by the healthcare system to provide these individuals with adequate care. Given that, in most cases, ASD requires lifelong support and treatment, one of the major challenges facing Brazil today is to expand the capacity of educational and healthcare services, in addition to training professionals to adequately serve this population. This includes expanding care availability and evaluating the quality and effectiveness of existing services.

In 2023, ASD accounted for 207,940 YLDs, being the sixth leading cause of YLDs among mental illnesses in Brazil, emphasizing the enormous impact of this condition on the country’s public health. A study published in 2023, using data from the Outpatient Health Care Registry (RAAS), found more than 1 million records related to care for children with ASD in Brazil^21^. In addition, there is strong evidence of an elevated risk of premature mortality in autistic individuals, with an increased risk of self-harm and suicide compared to the general population^22^. The presence of comorbid conditions and elevated mortality risk negatively affects the health of autistic individuals, and GBD Study 2023 burden estimates cannot yet quantify this^23^.

In the present study, ASD was more prevalent in men than in women and, in 2023, affected 0.82% of men and 0.25% of women, as in other studies^24^ ^,^ ^25^. A cross-sectional study associated with a cohort in southern Brazil found that the prevalence of ASD was around 0.3%, being more prevalent in boys (0.7% of boys) than in girls (0.1% of girls)^6^. Emerging evidence suggests that the higher prevalence of autism in individuals assigned male at birth compared to individuals assigned female at birth results from biological factors and identification biases^2^.

On the other hand, although less common, autism in girls tends to be more severe. A cross-sectional study conducted in the US analyzed population-based surveillance data from the Autism and Developmental Disabilities Monitoring Network for 20,135 8-year-old children with autism during the period from 2000 to 2016 to identify the prevalence and characteristics of individuals with profound autism (support level 3). The study found that among 8-year-old children with autism, 26.7% had this level of severity. In addition, children with level 3 support were more likely to be female, belong to racial and ethnic minority groups, have low socioeconomic status, and be born prematurely or with low birth weight^25^. The study observed that the prevalence of autism decreases in older age groups, since the GBD Study considers that ASD is present at birth, even when the diagnosis is delayed. This can be explained by the fact that, although the prevalence of ASD is higher in younger age groups, especially among children under 5 years of age, many studies still focus predominantly on the impact of the condition during childhood. Considering that some individuals receive a late diagnosis, this data overestimates the prevalence in children. As a result, healthcare services are better equipped to serve this age group. Nevertheless, care and support may extend into adolescence or adulthood^14^.

This study found that the prevalence of ASD, when age standardized, remained stable in Brazil between 1990 and 2023. It is important to note that the GBD Study framework is informed by a wide range of international studies used to construct the global model. However, when generating Brazil-specific estimates, the model relied on two Brazilian studies only, one from 2013 and the other from 2015^6^ ^,^ ^26^. Therefore, the 1990 estimates were not affected by the limited availability of ASD diagnoses in Brazil at that time.

Furthermore, a systematic review published in 2013 that included 49 articles from 1966 to 2012 highlighted that the prevalence of ASD already showed great variation between studies and between countries, ranging from 4 to 120 per 10,000 individuals, concluding that inconsistencies in the findings emphasize the importance of adopting comparable methods for the identification and estimation of ASD in order to ensure more consistent and accurate results^27^.

This study deepens and systematizes ASD estimates in the country, providing a more detailed and comprehensive analysis within the context of a developing nation of continental dimensions. Considering the existence of a universal healthcare system, which requires evidence-based planning to meet the needs of a large and diverse population, the findings contribute to a more qualified understanding of ASD prevalence, incidence, and YLDs. In this way, the study expands the available knowledge and provides relevant evidence to support the planning and organization of healthcare services at the national level. However, this study has some limitations: only two data sources for Brazil (the only country with representative studies in South America) were used, and the information presented is mainly based on modeling. Thus, we were unable to capture all the variation in ASD prevalence and burden among Brazilian regions, which were often presented within broad and overlapping limits of uncertainty. On the other hand, this is a robust modeling, periodically reviewed to incorporate additional data sources and update estimates for the country. Thus, more studies should be conducted with a representative population of the country, using culturally adapted and validated ASD diagnostic tools that are suitable for our population. Additionally, the data sources only include childhood diagnoses. Nevertheless, with meta-regression tools, it is possible to borrow the strength of data from data-rich locations to estimate prevalence in locations with little data available. This supports planning and management of healthcare policies, so that these estimates are, to date, the best available evidence of ASD prevalence, incidence, and YLDs for the country.

In 2023, Brazil had 1.1 million people with autism, accounting for 0.53% of the population and totaling more than 207,000 YLDs. The prevalence of ASD was higher in males and children under five years of age. Given that ASD is a lifelong condition and treatment is complex, it is important to recognize the significant burden of ASD in Brazil. Healthcare and education services at all levels must be adequately prepared to assist these individuals at all stages of life. In addition, further studies are needed to understand ASD morbidity in different states, based on primary research, as well as to validate screening and diagnostic tools specific to the Brazilian sociocultural context.

## References

[1] American Psychiatric Association (2021). Diagnostic and Statistical Manual of Mental Disorders (DSM-5).

[2] Lai MC, Amestoy A, Bishop S, Brown HM, Onaiwu MG, Halladay A (2023). Improving autism identification and support for individuals assigned female at birth: clinical suggestions and research priorities. Lancet Child Adolesc Health.

[3] Kuo SS, van der Merwe C, Fu JM, Carey CE, Talkowski ME, Bishop SL (2022). Developmental variability in autism across 17 000 autistic individuals and 4 000 siblings without an autism diagnosis: comparisons by cohort, intellectual disability, genetic etiology, and age at diagnosis. JAMA Pediatr.

[4] Bougeard C, Picarel-Blanchot F, Schmid R, Campbell R, Buitelaar J (2021). Prevalence of autism spectrum disorder and comorbidities in children and adolescents: a systematic literature review. Front Psychiatry.

[5] World Health Organization (WHO) Autism.

[6] La Maison C, Munhoz TN, Santos IS, Anselmi L, Barros FC, Matijasevich A (2018). Prevalence and risk factors of psychiatric disorders in early adolescence: 2004 Pelotas (Brazil) birth cohort. Soc Psychiatry Psychiatr Epidemiol.

[7] Girianelli VR, Tomazelli J, Silva CMFP, Fernandes CS (2023). Early diagnosis of autism and other developmental disorders, Brazil, 2013-2019. Rev Saude Publica.

[8] Buescher AVS, Cidav Z, Knapp M, Mandell DS (2014). Costs of autism spectrum disorders in the United Kingdom and the United States. JAMA Pediatr.

[9] Guze SB (1995). Diagnostic and Statistical Manual of Mental Disorders, 4th ed. (DSM-IV). Am J Psychiatry.

[10] World Health Organization (WHO) The ICD-10 classification of mental and behavioural disorders: clinical descriptions and diagnostic guidelines.

[11] Ferrari AJ, Santomauro DF, Aali A, Abate YH, Abbafati C, Abbastabar H (2024). Global incidence, prevalence, years lived with disability (YLDs), disability-adjusted life-years (DALYs), and healthy life expectancy (HALE) for 371 diseases and injuries in 204 countries and territories and 811 subnational locations, 1990-2021: a systematic analysis for the Global Burden of Disease Study 2021. Lancet.

[12] Brazilian Institute of Geography and Statistics (IBGE) IBGE - Brazilian Institute of Geography and Statistics.

[13] Shaw KA, Williams S, Patrick ME, Valencia-Prado M, Durkin MS, Howerton EM (2025). Prevalence and early identification of autism spectrum disorder among children aged 4 and 8 years - Autism and Developmental Disabilities Monitoring Network, 16 sites, United States, 2022. MMWR Surveill Summ.

[14] Paula CS, Cukier S, Cunha GR, Irarrázaval M, Montiel-Nava C, Garcia R (2020). Challenges, priorities, barriers to care, and stigma in families of people with autism: similarities and differences among six Latin American countries. Autism.

[15] Tromans S, Brugha T (2022). Autism epidemiology: distinguishing between identification and prevalence. Prog Neurol Psychiatry.

[16] Durkin MS, Elsabbagh M, Barbaro J, Gladstone M, Happe F, Hoekstra RA (2015). Autism screening and diagnosis in low-resource settings: challenges and opportunities to enhance research and services worldwide. Autism Res.

[17] World Health Organization Regional Office for South-East Asia (2014). Comprehensive and coordinated efforts for the management of autism spectrum disorders (ASD) and developmental disabilities (SEA/RC65/R8).

[18] World Health Organization Regional Office for South-East Asia (2012). Comprehensive and coordinated efforts for the management of autism spectrum disorders (ASD) and developmental disabilities (SEA/RC65/R8).

[19] Taylor B, Jick H, MacLaughlin D (2013). Prevalence and incidence rates of autism in the UK: time trend from 2004-2010 in children aged 8 years. BMJ Open.

[20] Wong VCN, Hui SLH (2008). Epidemiological study of autism spectrum disorder in China. J Child Neurol.

[21] Tomazelli J, Girianelli VR, Fernandes CS (2023). Incidence of pervasive developmental disorders in children: characteristics and analysis based on CAPSi data. Psicol USP.

[22] Santomauro DF, Hedley D, Sahin E, Brugha TS, Naghavi M, Vos T (2024). The global burden of suicide mortality among people on the autism spectrum: a systematic review, meta-analysis, and extension of estimates from the Global Burden of Disease Study 2021. Psychiatry Res.

[23] Santomauro DF, Erskine HE, Herrera AMM, Miller PA, Shadid J, Hagins H (2024). The global epidemiology and health burden of the autism spectrum: findings from the Global Burden of Disease Study 2021. Lancet Psychiatry.

[24] Montiel-Nava C, Peña JA (2008). Epidemiological findings of pervasive developmental disorders in a Venezuelan study. Autism.

[25] Hughes MM, Shaw KA, DiRienzo M, Durkin MS, Esler A, Hall-Lande J (2023). The prevalence and characteristics of children with profound autism, 15 sites, United States, 2000-2016. Public Health Rep.

[26] High-risk cohort study for psychiatric disorders in childhood: rationale, design, methods and preliminary results. PubMed.

[27] Isaksen J, Diseth TH, Schjølberg S, Skjeldal OH (2013). Autism spectrum disorders - are they really epidemic?. Eur J Paediatr Neurol.

