# Supplementary material for: Prevalence, incidence, and years lived with disability due to Autism Spectrum Disorder in Brazil, 1990-2023: Results from the Global Burden of Disease Study 2023
Source: Rev Bras Epidemiol. 2026 Aug 7;29:e260025. doi: 10.1590/1980-549720260025 (PMC13456913; doi:10.1590/1980-549720260025)
Supplement: Supplementary file 1 [file 1980-5497-rbepid-29-e260025-sppl.pdf]

Supplementary Material

Table S1. Disability weight attributed to ASD and its comorbidities by the Global Burden of Disease 2023.

Health condition	Lay description	Disability weight (II 95%)
Autism	Has difficulty interacting with other people and difficulty understanding simple questions and instructions. Difficulty with basic daily activities and changes in routine.	0.262 (0.176 - 0.365)
Asperger syndrome and ASD	Has difficulty interacting with others and takes a long time to understand or respond to questions. The person is often preoccupied with something and has difficulty with daily activities.	0.104 (0.071 - 0.147)
Borderline intellectual disability	Slow learner at school. As an adult, has some difficulty performing complex tasks, but demonstrates independence.	0.011 (0.005 - 0.020)
Mild intellectual disability	They have low intelligence and difficulty learning at school. As adults, they can live independently, but often need help raising children and can work if supervised.	0.043 (0.026 -0.064)
Moderate intellectual disability	They have low intelligence and are slow to learn to speak and even perform simple tasks. As an adult, they require a lot of support to live independently and raise children. They can only work in supervised and simpler jobs.	(0.100 (0.066 - 0.142)
Severe intellectual disability	Has very low intelligence. Cannot speak more than a few words. Needs constant supervision and assistance in most daily activities, although can perform the simplest tasks.	0.160 (0.107 - 0.226)
Profound intellectual disability	Has very low intelligence. Has almost no language skills and does not understand even simple requests and instructions. The person requires constant help for all activities.	0.200 (0.133 - 0.283)

Table S2. Characteristics of Brazilian studies used to estimate the burden of Autism Spectrum Disorder in Brazil between 1990 and 2023.

Authors	Location	Area	Age	Instruments	Sample size	Percentage of men	Metrics	Year
La Maison C et al[6]	Pelotas (RS)	Urban	11 years	DAWBA	3562	52	Prevalence	2015
Salum GA et al[26]	Porto Alegre (RS) e São Paulo (SP)	Urban	6 to 12 years	FHS; DAWBA	2512	53.1	Prevalence	2013
